# Research on the coordinated development of provincial urbanization and carbon emission efficiency of construction industry in China

**DOI:** 10.1186/s13021-024-00259-z

**Published:** 2024-04-13

**Authors:** Jianguang Niu, Boxiong Xin, Boyu Xin, Yuke Zhang, Mingqi Wang

**Affiliations:** 1 School of Urban Geology and Engineering, Hebei GEO University, Shijiazhuang, 050031 Hebei China; 2 Hebei Technology Innovation Center for Intelligent Development and Control of Underground Built Environment, Shijiazhuang, 050031 Hebei China; 3 Library, Hebei College of Resources and Environment Technology, Shijiazhuang, 050011 Hebei China

**Keywords:** Urbanization, CEECI, Coordinated development, Provincial differences, Optimization path

## Abstract

**Background:**

Energy conservation and emission reduction policies restrict the economic and social development of all countries in the world, and the impact on China, which has low urbanization, is more serious. In the process of promoting urbanization, the pressure of carbon emission reduction in the construction industry has increased, and the high emissions of the construction industry have made the low-carbon development of cities face severe challenges. China is at a critical stage of urbanization development, and there is become a general consensus on how to improve the carbon emission efficiency of the construction industry. The interaction between urbanization and the carbon emission efficiency of the construction industry is a long-term and complex process. As one of the industries contributing to China’s urbanization process and carbon emissions, it is of great practical significance to explore the coordination relationship between urbanization and the carbon emission efficiency of the construction industry (CEECI) to realize the goal of “double carbon”, promoting urbanization construction and solving the problem of “green development”. Taking 30 provinces in China as the research target area, the double weighted summation method and the undesirable output superefficiency window-EBM-DEA model are used to measure the provincial urbanization level and CEECI, respectively. Then, the coupling coordination degree model of the relative development index is introduced, and the spatial autocorrelation model and the spatial and temporal differentiation characteristics of the coordination level of urbanization and the CEECI are analysed.

**Results:**

From 2010 to 2021, China’s urbanization level increased steadily, but the growth rate gradually decreased. There were significant differences in urbanization levels among provinces. The eastern provinces have a higher level of urbanization but lack an impetus in the later period, while the western provinces have a lower level of urbanization but a faster growth rate. The low-carbon development trend of China’s construction industry is good, and the overall development of the CEECI shows an “N” type, and the inflection points appear in 2013 and 2018. The interprovincial urbanization level is significantly different from that of the CEECI, and the development form of the central and western provinces is better than that of the eastern provinces. The coordination level of urbanization and the CEECI in China is transitioning from the running-in stage to the coordination stage, and the coupling coordination degree between systems is on the rise, while the relative development degree is on the decline. The spatial distribution pattern is in a dynamic state of change, and the overall distribution pattern is “high in the east and low in the central and western regions”. The differences among provinces were significantly decreased, with 63.33% of provinces at the high running-in level. The provinces that entered the coordination stage were mainly located in the eastern region, and only Beijing was in the coordination stage by the end of the study. In addition, 90% of the provinces exhibited lagging efficiency, and only Jiangxi, Guangxi and Chongqing, which had low coordination levels maintained synchronous development of the two systems. The coordination level between provincial urbanization and the CEECI showed a positive spatial distribution, the global Moran index showed a “V” shape trend, and the spatial dependence of the coordination level between the two systems gradually weakened. In the local spatial distribution, there are two types of convergence: high and low.

**Conclusions:**

The coordination degree of urbanization and the CEECI analysed in this study is an extension of the research on the relationship between the two. By integrating the two into a unified framework, the method of combining quantitative and qualitative analysis is used to further explore the coordination relationship between the two, which not only enhances the scientificity and accuracy of the research but also extends the breadth and depth of relevant theoretical research. At the same time, according to the coordination type between urbanization and the CEECI, China should propose corresponding targeted coordination and optimization paths from the perspective of urbanization and low-carbon development of the construction industry to achieve high-quality development of China’s economy and society.

**Supplementary Information:**

The online version contains supplementary material available at 10.1186/s13021-024-00259-z.

## Introduction

With the development of economic and social modernization, climate warming has become a major global problem. The increasingly serious greenhouse effect poses a serious threat to the survival and development of human society. As the largest developing country, China is one of the world’s major carbon source contributors. China’s low-carbon development is crucial to global environmental governance. Therefore, to actively practice the concept of green development and fulfil carbon emission reduction obligations, China has proposed a series of energy conservation and emission reduction measures. In September 2020, China first proposed the “double carbon” target, that is, “strive to peak carbon dioxide emissions by 2030 and strive to achieve carbon neutrality by 2060” [1], and then further clarified in the “14th Five-Year Plan” that “energy consumption per unit of GDP and carbon dioxide emissions will be reduced by 13.5% and 18%, respectively’’ [2], marking China’s entry into the critical period of energy conservation and emission reduction. The number of cities and towns in China is increasing, with the urbanization rate increasing from 49.68% in 2010 to 64.72% in 2021. While China’s urbanization level is rapidly increasing, it also emits a large amount of carbon dioxide to the atmosphere. Studies have shown that 85% of China’s carbon emissions come from cities and towns [3], and properly addressing the relationship between urbanization and the environment has become an important issue. At the same time, urbanization is closely related to the construction industry. The construction industry, which has high energy consumption and high emissions, is the pillar industry of China’s economy and contributes greatly to the rapid development of China’s urbanization. The population agglomeration, lifestyle innovation and improvement of living standards driven by urbanization development have expanded the demand space for building consumption and will drive the development of the construction industry. This is bound to increase the demand for cement, steel and related energy, resulting in a large amount of carbon dioxide emissions and the continuation of construction industry carbon emissions. Carbon emissions from the manufacturing process of building materials (steel and cement, etc.) and fossil energy use in residential and commercial buildings contribute 9% of global direct carbon emissions, and electricity and heat use contribute 19% of indirect carbon emissions [4]. The high emissions of the construction industry will also restrict the high-quality development of urbanization. To improve the CEECI and encourage the urbanization process, as well as to support the high-quality coordinated development of urbanization and the construction industry, it is crucial to accurately measure the level of urbanization and the CEECI in China’s provinces and to analyse the coupling and coordination relationship between the two.

### Literature review

At present, scholars have produced abundant research results on urbanization and carbon emission efficiency.

The first is the study of urbanization. The research methods for evaluating urbanization mainly include the single evaluation index method and comprehensive evaluation index method. The single evaluation index method uses only one index to reflect the urbanization level, which is usually represented by the population urbanization rate [5]. Urbanization is a complex system, involving all aspects of economic and social development. The use of a single index is one-sided, and it is difficult to fully reflect the connotation of urbanization, which can easily lead to the loss of information. Therefore, to measure the level of urbanization more accurately, scholars has begun to use comprehensive indicators. Klauke studied urbanization by constructing an evaluation index system from four dimensions: population, occupation, living environment and distance from urban [6]. He Jing constructed the evaluation index system of urbanization from five dimensions of population, economy, land, society and ecology [7]. The measurement models used by the comprehensive index method can be divided into subjective and objective methods. Subjective methods mainly rely on expert experience for empowerment, and the measurement results are easily affected by subjectivity; therefore, scholars mostly use objective methods for empowerment. The objective methods used by scholars include the weighted kernel density method [8], factor analysis method [9] and entropy method [10]. The entropy method is mostly used in studies, but it has the defect of failing to reflect the time evolution trend of variables. Therefore, based on the comprehensive evaluation index, this paper uses the entropy method and the coefficient of variation method of increasing the time variable to jointly measure the urbanization level, which not only compensates for the defects of the entropy method but also reduces the possibility of bias in the results caused by the single weighting method.

The second is the study of carbon emission efficiency. The evaluation of carbon emission efficiency is divided into single factor efficiency and total factor efficiency evaluation. The single factor efficiency evaluation takes the ratio of carbon emissions to a certain factor as the evaluation standard. In 1993, Kaya and Yokobori first proposed “carbon productivity”, that is, the ratio of GDP per unit time to the total carbon dioxide emissions in the production process [11]. Carbon emission efficiency evaluations at the single factor level ignore the substitution effect among various production factors, and scholars gradually began to use the total factor efficiency evaluations. Therefore, the research on the CEECI is mostly based on the perspective of total factors, the calculation method is mostly data envelopment analysis, and the radial or nonradial DEA model is mainly used. Charnes and Cooper proposed a radial DEA model suitable for multi-input‒output indicators and applied it in CEECI evaluation [12]. For the first time, Zaim et al. took carbon emissions as an undesirable output and used the radial DEA model to measure carbon emission efficiency [13]. Tone established a nonradial slacks-based measure (SBM) model to determine the impact of relaxation variables in outputs on efficiency measurements [14]. Hui et al. used the nonradial superefficiency SBM-DEA model to calculate the CEECI and analyse the characteristics of the Chinese CEECI [15].

Third, the relationship between urbanization and carbon emission efficiency is studied. At present, the long-term equilibrium relationship between urbanization and carbon emission efficiency has been confirmed by many studies. Li et al. believe that urbanization can promote carbon emission efficiency, but the intensity of the impact is different in different regions, and the promotion effect is the strongest in western China [16]. According to Sun et al., there is an inverted U-shaped relationship between urbanization and carbon emission efficiency. Once the urbanization level crosses the critical point, urbanization will have an inhibitory effect on carbon emission efficiency [17]. With further research, scholars’ research on carbon emission efficiency and urbanization has been further refined at specific industry levels. For example, Liu et al. found that urbanization has a low promotion effect on the carbon emission efficiency of the tourism industry, but the promotion effect of urbanization gradually increases with time [18]. Zhao et al. showed that the urbanization level and urban population density had a significant negative impact on the CO_2_ emission efficiency of the transportation sector [19]. At the same time, the high energy consumption and high emissions of the construction industry make the construction industry have made this industry a concern of scholars. For example, Chen used the spatial lag model to show that an increase in the urbanization rate can promote an increase in the CEECI [20].

In summary, although there are many studies on urbanization and the CEECI, the existing studies mainly analyse urbanization as a factor affecting the CEECI, and few studies explore the coordination degree of the two from the perspective of urbanization. Second, there are some defects in the research on urbanization evaluation indicators and evaluation methods: (1) the index system of high-quality development of urbanization is not perfect, and there are few indicators that can reflect high-quality development; (2) the determination of indicator weights in the evaluation system mainly relies solely on expert experience or historical data, which causes the single weighting method to have certain drawbacks. Finally, the study of the CEECI does not avoid the influence of the radial and nonradial defects of DEA on the CEECI. Moreover, the input and output data used by the classical DEA model are the cross-sectional data from the current year, and only the influence of inputs on the output of the current year can be calculated; thus, dynamic studies on DMUs cannot be carried out on time series.

### Research contributions and purposes

By combing the literature, the current research on urbanization and the CEECI is mostly limited to the analysis of the interaction between the two, and there are few studies on the coordinated development between the two. At the same time, the evaluation of the urbanization level and CEECI has the disadvantages of one-sidedness and low accuracy. Therefore, the contributions of this paper are as follows: (1) the urbanization and the CEECI are two subsystems used to explore the degree of coordinated development between the two subsystems; (2) the evaluation index system is constructed from the multidimensional connotation of urbanization, and the double objective weighting method is used to measure the urbanization level to avoid the disadvantages of the single weighting method and subjective weighting method; and (3) on the basis of constructing the undesirable output superefficiency EBM model, the Windows-DEA model is introduced to avoid the disadvantages of traditional radial and nonradial models while making the CEECI comparable in the vertical direction.

This paper aims to answer three questions: (1) What are the respective development levels of urbanization and the CEECI in China’s provinces? (2) Are the two in a state of coordinated development, and what is the degree of coordination? (3) How can high-quality coordinated development of the two be achieved? Answering the above questions will provide a theoretical basis for the formulation of high-quality coordinated development policies for China’s provincial urbanization and construction industry.

This paper will address the appeals question from five different perspectives. That is, the first chapter puts forwards the research contribution and purpose of this paper through the introduction of the research background and literature review. The second chapter describes the evaluation index, and the research methods and models are discussed. The third chapter analyses the measurement results of the urbanization level and CEECI in 30 provinces of China. The fourth chapter analyses the three dimensions of the coordination level of urbanization and the CEECI in 30 provinces of China: spatial and temporal evolution trends, spatial correlation characteristics and optimized development paths. The fifth chapter is a summary of the previous article and presents the research conclusions and research deficiencies.

## Index system and method

### Study area: China

The study area of this paper is China’s 34 subprovincial administrative divisions. Due to differences in statistical standards, Hong Kong, Macau, and Taiwan, as well as Tibet due to some missing statistical data, were not included in the study scope. Therefore, the target area of this study is 30 provinces in China from 2010 to 2021.

**Fig.1:**
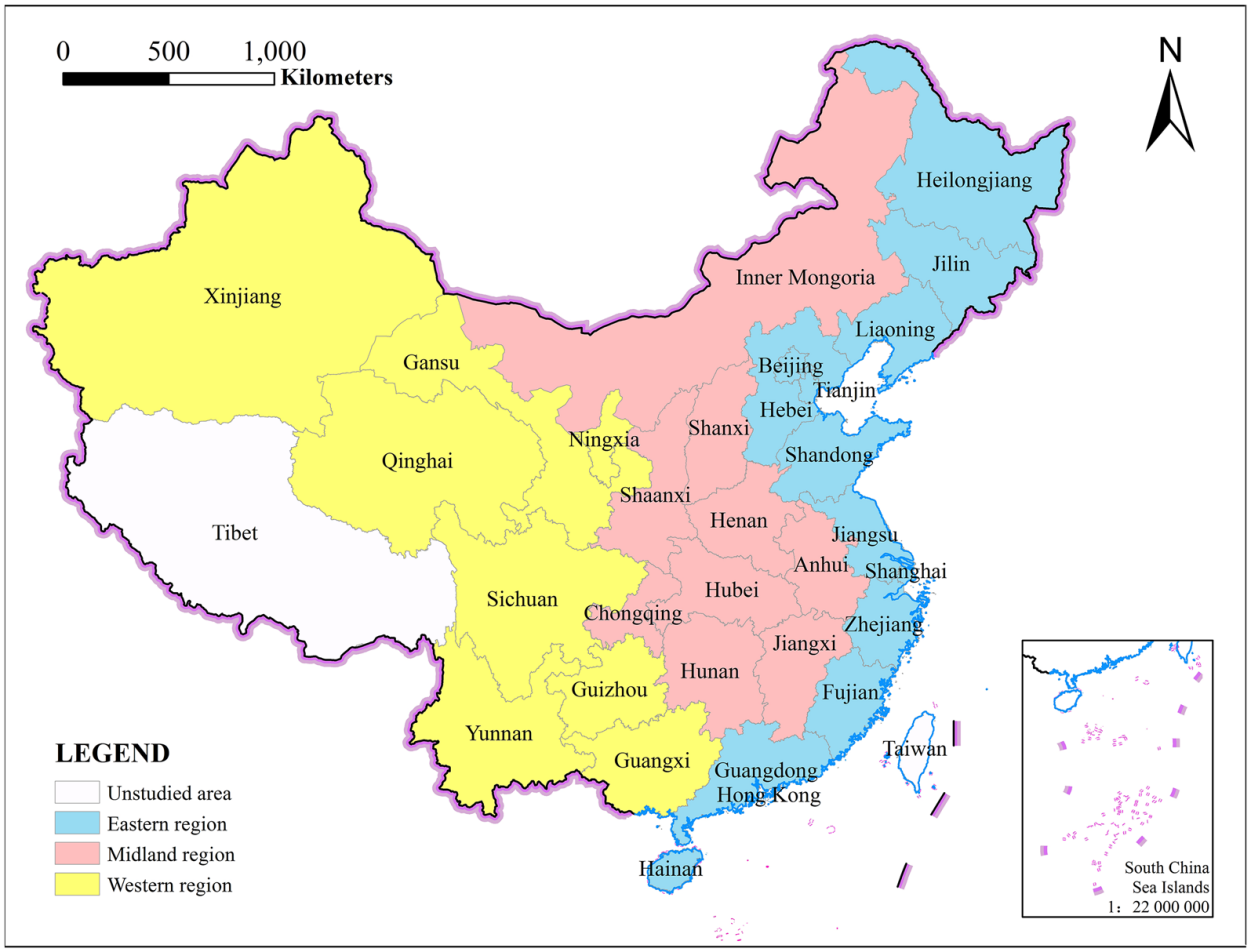
Regional division

Current research divides the country into three regions—the eastern, central, and western regions—based on regional and geographical factors. Thirteen provinces and cities, including Beijing, Tianjin, Shanghai, Heilongjiang, Jilin, Liaoning, Hebei, Shandong, Jiangsu, Zhejiang, Fujian, Guangdong, and Hainan, compose the eastern region of China, which resides mainly in the country's eastern coastal areas. The aforementioned locations have high rates of urbanization and population aggregation due to their high levels of openness and rapid economic development. This further promotes the growth of the construction industry and results in substantial greenhouse gas emissions. The central region’s economic and urbanization growth has been relatively reasonable, and the development of the building industry, which includes Inner Mongolia, Shanxi, Hunan, Shaanxi, Hubei, Chongqing, Anhui, Hunan, Jiangxi and nine other provinces and cities, is likewise sluggish. Due to their low economic volume and sparse population, the provinces in the western region have insufficient urbanization development potential, which further restricts the development of the construction industry. However, the ecological environment in the western region is good, and this region, which includes Xinjiang, Gansu, Qinghai, Ningxia, Sichuan, Guizhou, Yunnan, Guangxi and other eight other provinces, has great potential for ecological urbanization and carbon balance. The provinces of each region are displayed in Fig. 1.

### Evaluation index system of the coordination level between urbanization and the CEECI

The evaluation index system of the coordination level of urbanization and the CEECI is the basis for evaluating the level of their respective dimensions and the degree of coordinated development of the two, which determines the accuracy and authenticity of the measurement results. Following the principles of objectivity, independence and data availability, the constructed coordination level evaluation system includes 2 subsystems and is divided into 36 specific indicators (Table 1).

**Table 1:** Evaluation index system of coordination level between urbanization and CEECI

Subsystem	Dimension	Index layer
Urbanization level	Population urbanization	Urbanization rate of permanent population
		Proportion of employed persons in the secondary industry
		Proportion of employed persons in the tertiary industry
		Urban population density
	Economic urbanization	GDP per capita
		The proportion of the secondary industry output value in GDP
		The proportion of the tertiary industry output value in GDP
		Per capita disposable income of urban residents
		Per capita fiscal revenue
	Land urbanization	Area of per capita urban
		Area of per capita built-up
		Area of per capita of construction land
		Area of per capita urban road
	Social urbanization	Registration unemployment rate of urban population
		Water popularization rate
		Gas popularization rate
		The number of buses per 10,000 people
		Health technicians per 1000 population
		Per 1000 people have beds in medical institutions
		Public library collections per capita
		per capita expenditure on education
		Total retail sales of social consumer goods
	Ecological urbanization	Energy consumption per ten thousand yuan GDP
		Green coverage rate of built-up zones
		Per capita park green area
		Harmless treatment rate of domestic waste
		Daily treatment capacity of urban sewage
	Urban and rural co-ordination	Comparison of urban and rural residents income level (urban/rural)
		Comparison of urban and rural residents consumption level (urban/rural)
CEECI	Input indicators	Construction industry fixed assets
		Number of construction industry employees
		Total power of its own construction machinery and equipment
		Energy consumption in construction industry
	Output indicators	Total output value of construction industry
		Building completion area
		Carbon emissions of construction industry

The urbanization evaluation index system is based on the “National New Urbanization Plan (2014–2020)” and other documents combined with the reality and characteristics of China’s urbanization development. Based on the research of HE Jing, Jiang Jikun and Wang Zhaofeng [7, 21, 22], this paper constructs the multidimensional connotation of new urbanization, including 6 dimensions: population, economy, land, society, ecological urbanization and urbanrural integration.

Referring to the research of scholars such as Liu H, Zhang GT and Song JZ [23–25], this paper defines the total factor carbon emission efficiency as the maximum economic output and the minimum carbon emission that can be achieved in the production process of the construction industry with the gradual increase in input and output. From the two dimensions of input and output, combined with correlation analysis, the CEECI evaluation index system is established. The input indicators include four aspects: labour, mechanical equipment, capital and energy input. The output indicators include two aspects: expected output (total output value of the construction industry and building completion area) and undesired output (carbon emissions of the construction industry).

### Data sources

Based on the availability of data, the data used in this paper on urbanization evaluation are from the “China Statistical Yearbook”, each of which includes provincial statistical yearbooks and statistical bulletins. The CEECI measurement data are derived from the “China Construction Industry Statistical Yearbook’’, “China Energy Statistical Yearbook”, “China Statistical Yearbook” and statistical yearbooks and statistical bulletins of various provinces and cities. At the same time, the missing values are filled by interpolation, and the abnormal values after the analysis of the relevant environment are eliminated and filled.

The carbon emission of the construction industry is based on the whole life cycle assessment method, and the carbon emission of the construction industry is determined to be the sum of the carbon emission of the building material production link, the carbon emission of the building material transportation link, the carbon emission of the construction link, the carbon emission of the later operation link and the carbon emission of the demolition link. The specific accounting process of carbon emissions in the construction industry is based on the research of Liu [26], and the recycling coefficient of building materials is based on the research of Cui [27].

### Method and model

#### Double weighted summation method

At present, the evaluation of urbanization is mostly based on the weighting of indicators, and the determination of index weights in the evaluation system is mainly based on expert experience or historical data, which causes a single weighting method to have certain drawbacks. Therefore, this paper uses the geometric average method to combine the improved entropy method [28] with the coefficient of variation method [29] to establish an urbanization level evaluation model. The formula is as follows:

1 $${U}_{\theta i}=\sqrt{{\sum }_{j=1}^{m}\left({w}_{j}\cdot {x}_{\theta ij}^{\prime}\right)\times {\sum }_{j=1}^{m}\left({\omega }_{j}\cdot {x}_{\theta ij}^{\prime}\right)}$$

In Formula (1), ${U}_{\theta i}$ denotes the urbanization level of area *i* in year *θ* year; ${w}_{j}$ is the weight of index *j* weighted by the improved entropy method; ${\omega }_{j}$ is the weight of the index *j* weighted by the coefficient of variation; and ${x}_{\theta ij}^{\prime}$ which represents the standardized data of index *j* in region *i* of year *θ*.

##### The improved entropy method

To account for its inability to capture the time evolution trend of variables, the improved entropy method introduces temporal variables based on the traditional entropy method. First, the original data are not dimensionalized using the range standardization approach. Equations (2) and (3) are used for the positive and negative indices, respectively. The following is the formula:

2 $${x}_{\theta ij}^{\prime}=\frac{{x}_{\theta ij}-{x}_{min}}{{x}_{max}-{x}_{min}}$$

3 $${x}_{\theta ij}^{\prime}=\frac{{x}_{max}-{x}_{\theta ij}}{{x}_{max}-{x}_{min}}$$

where ${x}_{\theta ij}$ for the original data, denotes the *j*th index value of the *i*th region in *θ* years and ${x}_{max}$ and ${x}_{min}$ represent the maximum and minimum values of the original data, respectively. Then, the index weight is determined by Formula (4).

4 $${y}_{\theta ij}=\frac{{x}_{\theta ij}^{\prime}}{\sum_{\theta =1}^{r}\sum_{i=1}^{n}{x}_{\theta ij}^{\prime}}$$

In Formula (4), *θ* denotes the year, a total of 12 years; *i* indicates the region, a total of 30 regions; and ${y}_{\theta ij}$ represents the weight of the *j*th index value of the *i*th region in year *θ*. The entropy value of the index is calculated by using Formula (5).

5 $${e}_{j}=-\frac{\sum_{\theta =1}^{r}\sum_{i=1}^{n}{y}_{\theta ij}{\text{ln}}\left({y}_{\theta ij}\right)}{{\text{ln}}rn}$$

In Formula (5), ${e}_{j}$ is the index entropy value, and 0 ≤ ${e}_{j}$≤1. The Formula (6) is used to determine the total weight of a single index.

6 $${w}_{j}=\frac{{g}_{j}}{\sum_{j=1}^{m}{g}_{j}} , {g}_{j}=1-{e}_{j}$$

In Formula (6), ${g}_{j}$ is the information utility value of the index, and* m* is the number of indices *j*.

##### Coefficient of variation method

The coefficient of variation method is based on the degree of variation between the current value and the target value of each evaluation index to weight each index. If the value of an index is quite different, it can clearly distinguish the evaluated objects, indicating that there is a wealth of resolution information in the index, so the index should be given a larger weight. In contrast, the opposite is true. First, the coefficient of variation of the index is calculated. Second, the index is weighted according to the coefficient of variation. Finally, the weighted summation method is used to calculate the new urbanization level. The specific formula is as follows:

7 $${V}_{j}=\frac{{S}_{j}}{{A}_{j}}$$

8 $${\omega }_{j}=\frac{{V}_{j}}{\sum_{j=1}^{m}{V}_{j}}$$

In Formula (7), ${V}_{j}$ is the coefficient of variation of the *j*th index, ${S}_{j}$ is the standard deviation of the *j*th index, and ${A}_{j}$ is the average value of the *j*th index.

#### CEECI measurement model

The radial DEA ignores the slack variables, resulting in inaccurate efficiency values; the projection point of the evaluated unit in the nonradial SBM model is the farthest point from the evaluated unit on the frontier, and based on the evaluation angle, the goal is to reach the frontier with the shortest path, which is contrary to each other. Therefore, referring to the practice of Zeng [30], this paper constructs a superefficiency EBM-DEA model considering undesirable outputs to measure CEECI, which combines radial and nonradial methods, as shown in Formula (9).

9 $$ \begin{array}{*{20}l} {\rho^{*} = \min \frac{{\theta - \varepsilon_{x} \mathop \sum \nolimits_{i = 1}^{m} \frac{{w_{i}^{ - } s_{i}^{ - } }}{{x_{ik} }}}}{{\varphi + \varepsilon_{y} \mathop \sum \nolimits_{r = 1}^{n} \frac{{w_{r}^{ + } s_{r}^{ + } }}{{y_{rk} }} + \varepsilon_{b} \mathop \sum \nolimits_{p = 1}^{q} \frac{{w_{p}^{b - } s_{p}^{b - } }}{{b_{pk} }}}}} \\ {s.t.\left\{ {\begin{array}{*{20}l} {\sum_{{j = 1,j \ne j_{0} }}^{k} x_{ij} \lambda_{j} + s_{i}^{ - } = \theta x_{ik} , \quad i = 1,2,3 \cdot \cdot \cdot m} \\ {\sum_{{j = 1,j \ne j_{0} }}^{k} y_{ij} \lambda_{j} - s_{i}^{ - } = \varphi y_{rk} , \quad r = 1,2,3 \cdot \cdot \cdot n} \\ {\sum_{{j = 1,j \ne j_{0} }}^{k} b_{pj} \lambda_{j} + s_{p}^{b - } = \varphi b_{pk} , \quad p = 1,2,3 \cdot \cdot \cdot q} \\ {\lambda_{j} \ge 0 , s_{i}^{ - } ,s_{r}^{ + } ,s_{p}^{b - } \ge 0 } \\ \end{array} } \right.} \\ \end{array} $$

In Formula (9), ${\rho }^{*}$ is the efficiency; *φ* is the output expansion ratio; *k* is the decision-making unit (*DMU*); *m* is the type of input; *n* is the type of expected output; *q* is the type of undesired output; *θ* is the radial partial programming parameter; ${\varepsilon }_{x}$ and ${\varepsilon }_{y}$ are the key parameters, satisfying 0 ≤  ${\varepsilon }_{x}$  ≤  1, 0 ≤  ${\varepsilon }_{y}$≤ 1; ${w}_{i}^{-}$ is the importance of input factors, and its sum is 1; ${x}_{ik}$ and ${y}_{rk}$ represent the *i* input and *r* output of the decision-making unit; ${s}_{i}^{-}$,${s}_{r}^{+}$ and ${s}_{p}^{b-}$ are relaxation variables; ${w}_{r}^{+}$ and ${w}_{p}^{b-}$ represent the weights of expected output and undesirable output, respectively; ${b}_{pk}$ is the type *p* undesirable output of the decision making unit; ${\lambda }_{j}$ is the linear combination coefficient; and ${j}_{0}$ represents the superefficiency value of ${DMU}_{j}$ on the new effective frontier excluding ${DMU}_{{j}_{0}}$ when the evaluated decision-making unit is ${DMU}_{{j}_{0}}$.

To sure that the efficiency values cannot be compared vertically, this paper introduces the Windows-DEA model, which uses the principle of a similar moving average to select the reference set. According to the existing research [31], the window width *d* of the Windows-DEA model is set to 3. Finally, the average efficiency of each year in different windows is used as the efficiency value that the DMU can finally compare vertically. Therefore, this paper finally uses the superefficiency Windows-EBM-DEA model considering undesired outputs to measure the CEECI.

#### Coordination level evaluation model

The coupling coordination degree model can not only describe the degree of correlation between the two systems but also make the coordinated development between the systems comparable. The modified coupling degree model proposed by Wang et al. [32] is used to construct the coupling coordination degree model. The specific formula is:

10 $$W=\sqrt{[1-\left({X}_{3}-{X}_{4}\right)]\times \frac{{X}_{4}}{{X}_{3}}}$$

11 $$T=\alpha {X}_{1}+\beta {X}_{2},\alpha +\beta =1$$

12 $$S=\sqrt{W\times T}$$

where $W$ is the coupling degree between the two systems; ${X}_{1}$ is the urbanization level; ${X}_{2}$ is the CEECI; ${ X}_{3}={\text{max}}\left\{{X}_{1},{X}_{2}\right\}{,X}_{4}={\text{min}}\left\{{X}_{1},{X}_{2}\right\}$; $T$ is the overall benefit level of the two subsystems; and $\alpha$ and $\beta$ are undetermined coefficients, which mainly reflect the contributions of the two subsystems to the overall benefit level. The results of the two systems are weighted by the improved entropy method. The results show that the weights of the two systems are both 0.5, which is consistent with the government’s equal attention to the new urbanization construction and the low-carbon development of the construction industry in recent years. Therefore, the sum is set to 0.5. $S$ is the coupling coordination degree of the two systems. The closer the value is to 1, the better the coupling and coordinated development of the urbanization level and the CEECI are.

The coupling coordination degree cannot reflect the relative development degree between systems, so the relative development degree model is introduced with reference to Sun et al. [33].

13 $$E=\frac{{X}_{2}}{{X}_{1}}$$

In Formula (13), $E$ is the relative development degree.

According to HE research [34], the equal distance method is used to divide the stages and types of the coordinated development of urbanization and CEECI coupling, as shown in Table 2.

**Table 2:** The coupling of the urbanization and CEECI coordinated development stage and type division standard

Coupling coordination degree/*S*	Development stage	Rank of harmony degree	Relative development degree/*E*	Coordinated development type
[0.9, 1]	Coordinating	Best coordination	*E* ≤ 2.8	Best coordination efficiency lag type
			2.8 < *E* ≤ 4.2	Best coordination synchronous type
			*E* > 4.2	Best coordinated urbanization lag type
[0.8, 0.9)		Good coordination	*E* ≤ 2.8	Good coordination efficiency lag type
			2.8 < *E* ≤ 4.2	Good coordination synchronous type
			*E* > 4.2	Good coordinated urbanization lag type
[0.7, 0.8)		Medium coordination	*E* ≤ 2.8	Medium coordination efficiency lag type
			2.8 < *E* ≤ 4.2	Medium coordination synchronous type
			*E* > 4.2	Medium coordinated urbanization lag type
[0.6, 0.7)		Low coordination	*E* ≤ 2.8	Low coordination efficiency lag type
			2.8 < *E* ≤ 4.2	Low coordination synchronous type
			*E* > 4.2	Low coordinated urbanization lag type
[0.5, 0.6)	Running-in	High running-in	*E* ≤ 2.8	High running-in efficiency lag type
			2.8 < *E* ≤ 4.2	High running-in synchronous type
			*E* > 4.2	High running-in urbanization lag type
[0.4, 0.5)		Low running-in	*E* ≤ 2.8	Low running-in efficiency lag type
			2.8 < *E* ≤ 4.2	Low running-in synchronous type
			*E* > 4.2	Low running-in urbanization lag type
[0.3, 0.4)	Antagonistic	Low antagonistic	*E* ≤ 2.8	Low antagonistic efficiency lag type
			2.8 < *E* ≤ 4.2	Low antagonistic synchronous type
			*E* > 4.2	Low antagonistic urbanization lag type
[0.2, 0.3)		Medium antagonistic	*E* ≤ 2.8	Medium antagonistic efficiency lag type
			2.8 < *E* ≤ 4.2	Medium antagonistic synchronous type
			*E* > 4.2	Medium antagonistic urbanization lag type
[0, 0.2)		High antagonistic	*E* ≤ 2.8	High antagonistic efficiency lag type
			2.8 < *E* ≤ 4.2	High antagonistic synchronous type
			*E* > 4.2	High antagonistic urbanization lag type

#### Spatial autocorrelation model

The spatial autocorrelation model is mainly used to test whether there is a spatial correlation between a certain area and its adjacent areas. At present, the methods used to study spatial correlation are mainly global Moran’s I and local Moran’s I. Global Moran’s I measures the spatial correlation of the entire study area. The formula is as follows:

14 $${I}_{g}=\frac{n\sum_{i=1}^{n}\sum_{j=1}^{n}{\omega }_{ij}\left({x}_{i}-\overline{x }\right)\left({x}_{j}-\overline{x }\right)}{\sum_{i=1}^{n}\sum_{j=1}^{n}{\omega }_{ij}{\sum }_{i=1}^{n}{\left({x}_{i}-\overline{x }\right)}^{2}}, \quad i,j=1,2,3\dots \dots n, i\ne j$$

In Formula (14), ${I}_{g}$ is the global Moran’s I; *n* is the number of provinces; ${x}_{i}$, and ${x}_{j}$ represent the coupling coordination degrees of provinces *i* and *j*, respectively; $\overline{x }$ represents the average value of the coupling coordination degree; and ${\omega }_{ij}$ is the economic distance weight matrix.

The local Moran’s I can further obtain the location of the aggregation, and the formula is as follows:

15 $${I}_{l}={Z}_{i}{\sum }_{j=1}^{n}{\omega }_{ij}{Z}_{j}, \quad i,j={1,2},3\dots \dots n, i\ne j$$

In Formula (15), ${I}_{l}$ is the local Moran’s I, and ${Z}_{i}$, and ${Z}_{j}$ are the standardized values of the coupling coordination degree of provinces *i* and* j*.

#### Study of the process framework of the model

**Fig.2:**
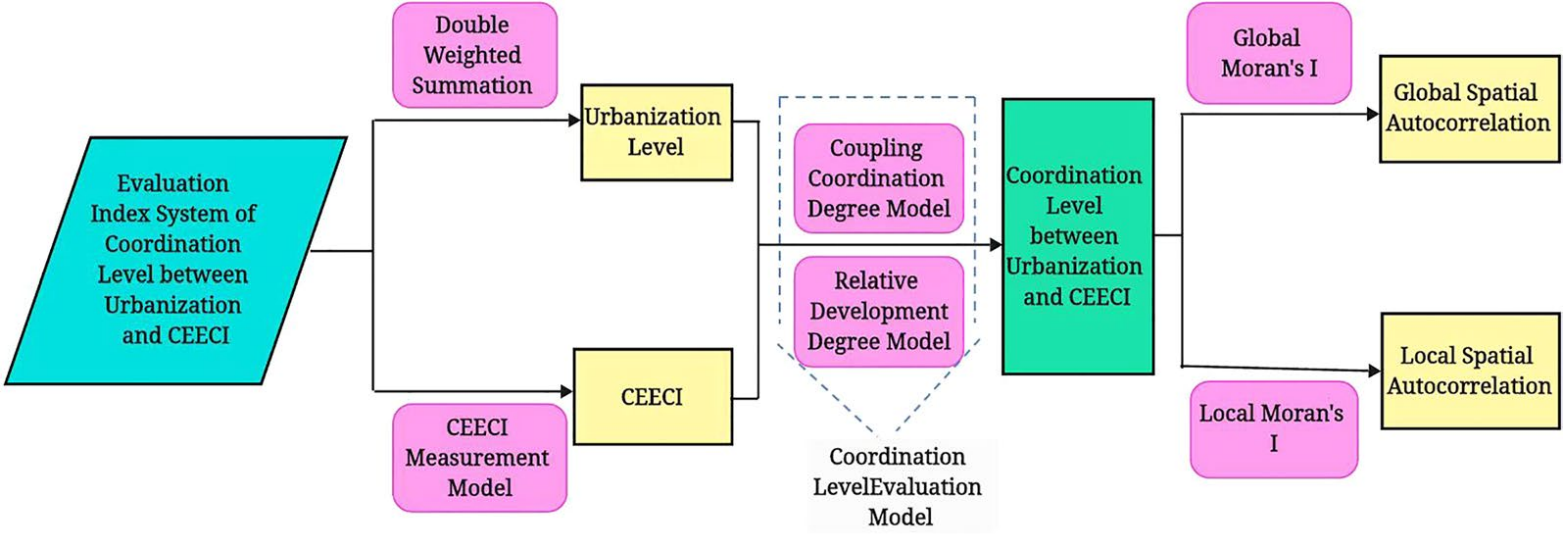
Study of the process framework of the model

The model process framework of this study is shown in Fig. 2. First, based on the panel data of 30 provinces in China from 2010 to 2021, the two subsystems of provincial urbanization and the CEECI are measured by the double weighted summation method (combined with the improved entropy method and coefficient of variation) and the CEECI measurement model (superefficiency Windows-EBM-DEA model considering undesired output). Then, the coordination level evaluation model (including the coupling coordination degree model and the relative development degree model) is used to evaluate the coordination level of the two subsystems. Finally, according to the evaluation results of the coordination level, the spatial autocorrelation analysis method (including global Moran’s I and local Moran’s I) is used to describe the spatial correlation characteristics.

## Urbanization level and CEECI evaluation

### Evaluation of provincial urbanization level

Due to space limitations, the results only list the beginning and ending years of the “5-year plan” during the study period (Table 3).

**Table 3:** Measurement results of China’s provincial urbanization level

Regional	Province	2010	2011	2015	2016	2020	2021	Average	Average annual growth rate (%)
East	Beijing	0.464	0.493	0.537	0.576	0.612	0.624	0.555	2.717
	Tianjin	0.290	0.323	0.382	0.407	0.439	0.455	0.383	4.178
	Hebei	0.192	0.207	0.259	0.273	0.326	0.337	0.266	5.243
	Liaoning	0.262	0.287	0.348	0.365	0.390	0.403	0.346	3.996
	Jilin	0.214	0.223	0.287	0.304	0.369	0.381	0.294	5.396
	Heilongjiang	0.201	0.219	0.298	0.313	0.359	0.370	0.294	5.715
	Shanghai	0.428	0.449	0.502	0.505	0.561	0.577	0.503	2.753
	Jiangsu	0.318	0.345	0.434	0.456	0.505	0.539	0.437	4.908
	Zhejiang	0.296	0.319	0.400	0.422	0.475	0.497	0.407	4.829
	Fujian	0.211	0.234	0.300	0.312	0.377	0.396	0.309	5.887
	Shandong	0.281	0.307	0.382	0.397	0.446	0.467	0.383	4.747
	Guangdong	0.305	0.325	0.403	0.426	0.490	0.513	0.411	4.830
	Hainan	0.191	0.243	0.308	0.302	0.369	0.368	0.298	6.164
Midland	Shanxi	0.164	0.181	0.255	0.267	0.318	0.333	0.254	6.685
	Inner Mongolia	0.210	0.313	0.313	0.329	0.362	0.368	0.317	5.237
	Anhui	0.171	0.191	0.270	0.280	0.371	0.387	0.279	7.703
	Jiangxi	0.179	0.199	0.252	0.264	0.336	0.356	0.265	6.486
	Henan	0.153	0.171	0.253	0.270	0.344	0.358	0.261	8.042
	Hubei	0.198	0.217	0.302	0.320	0.378	0.393	0.305	6.409
	Hunan	0.168	0.182	0.248	0.266	0.353	0.371	0.262	7.452
	Shaanxi	0.162	0.182	0.256	0.266	0.315	0.341	0.254	7.015
	Chongqing	0.180	0.217	0.302	0.315	0.354	0.375	0.299	6.905
West	Sichuan	0.155	0.178	0.264	0.283	0.363	0.379	0.273	8.438
	Guizhou	0.092	0.114	0.207	0.228	0.303	0.303	0.213	11.413
	Yunnan	0.118	0.133	0.201	0.226	0.278	0.298	0.209	8.817
	Guangxi	0.172	0.179	0.245	0.259	0.327	0.339	0.256	6.338
	Gansu	0.118	0.137	0.226	0.242	0.301	0.312	0.226	9.243
	Qinghai	0.144	0.160	0.223	0.236	0.326	0.333	0.235	7.957
	Ningxia	0.282	0.288	0.344	0.349	0.371	0.368	0.341	2.442
	Xinjiang	0.214	0.242	0.315	0.325	0.365	0.376	0.309	5.229
Nationwide		0.218	0.242	0.311	0.326	0.383	0.397	0.315	5.617

**Fig. 3:**
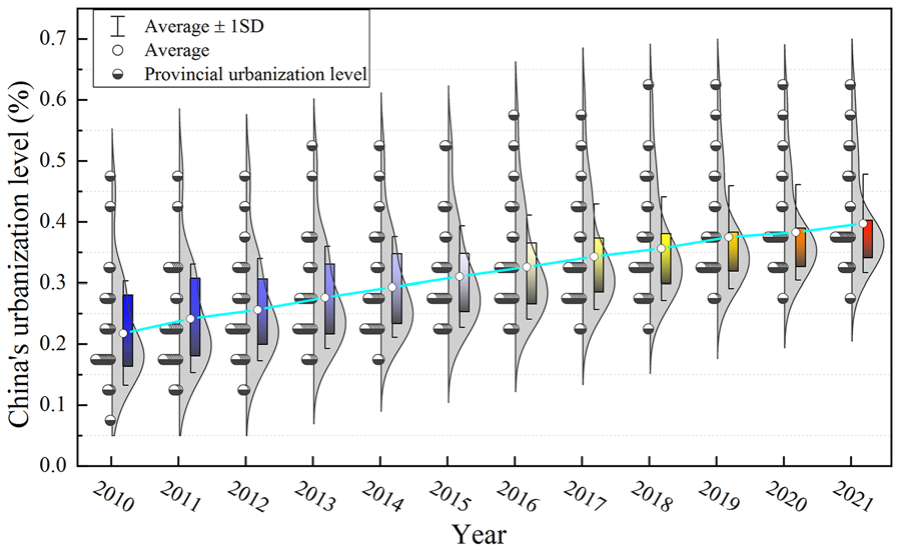
China’s urbanization level violin figure

From Table 3 and Fig. 3 show that China’s urbanization level is steadily increased from 2010 to 2021, from 0.218 to 0.397, with an average annual growth rate of 5.617%. However, the year-on-year growth rate of China’s urbanization level shows a fluctuating downwards trend, from 11.469% to 3.783%, indicating that China’s urbanization development has achieved certain results, but the later development vitality is insufficient, and policy stimulation needs to be further increased. See in stages, During the “12th Five-Year Plan” period, China’s urbanization level rapidly improved, with an average annual growth rate of 7.356%. The realization of rapid growth is mainly due to the middle government’s proposal to accelerate the transformation of the urbanization development mode and put forward a new type of urbanization with people as the core, which further increase the development potential of urbanization. In the “13th Five-Year Plan’’ period, the level of urbanization continued to improve, but the rate of improvement tended to decrease, with an average annual increase of 4.272%, indicating that with increasing urbanization, the positive effect of previous policies on this period has gradually weakened. It is necessary to formulate new policies to realize the innovation of the urbanization development mode and inject new vitality into urbanization development.

From the perspective of the form of the violin map (Fig. 3), the level of urbanization in China’s provinces is significantly different, and it is mainly concentrated in low-value areas. The cabinet in the violin diagram is significantly shortened, indicating that the differentiation characteristics of China’s urbanization level have weakened, and the tail of the upper end has gradually lengthened, indicating that the provinces in the high-value areas have gradually increased. This is due to the implementation of active regional support policies in China, which has gradually weakened the differences in urbanization levels among provinces. It is necessary to further guide and strengthen the radiation effect of high-value provinces on low-value provinces.

From a regional perspective, the level of new urbanization in China from 2010 to 2021 presented an overall distribution pattern of “higher in the east, followed by the central part and lower in the west”. The growth trend is consistent with the national average annual growth rates of the eastern, central and western regions is 4.115%, 6.115% and 6.175%, in the eastern, central and western regions, respectively, showing that “the western region is faster, followed by the central region and the eastern region is slower”. The rapid growth of the western region is mainly due to the implementation of strategies and policies such as the development of the western region, while the development of new urbanization in the eastern region has entered a bottleneck period due to its high economic and social level, and it is difficult to rapidly improve. This shows that the new-type urbanization level of provinces and cities has a positive relationship with regional geographical location and regional economic development level.

**Fig.4:**
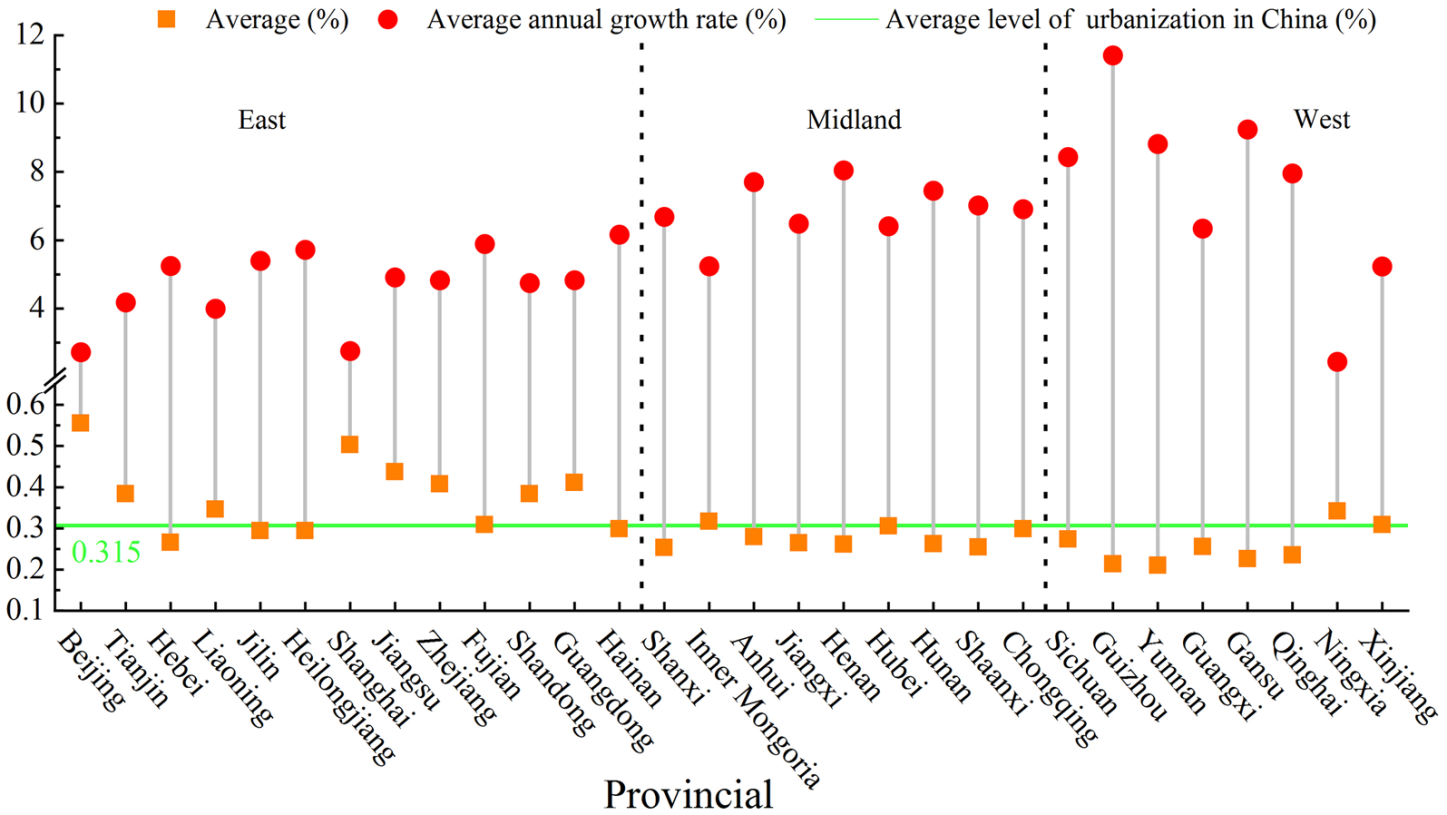
The average level and average annual growth rate of urbanization in China’s provinces

At the provincial level (Fig. 4), there are 10 provinces whose average urbanization level exceeds the national average of 0.315, 8 provinces are located in the eastern region, mainly municipalities or located in the southeastern coastal areas, and the urbanization level is generally above 0.400. Beijing and Shanghai city have a good foundation for urbanization construction due to their rapid economic and social development, which results in urbanization level above 0.5 in the two regions. Only one autonomous region in Inner Mongolia is located in the central region, and its urbanization construction effect is remarkable, mainly because it has vast land and rich resources, which provides good conditions for the development of urbanization. A series of policy documents on the large-scale development of agriculture and animal husbandry were issued and implemented so that farmers and herdsmen gradually gathered in cities and towns. These conditions promote the process of urbanization in different ways. In the western region, only one province in Ningxia had a greater level of urbanization than the national average, and the level of urbanization during the study period was greater than that of other northwestern provinces, which is consistent with the result of factor analysis in Xu Ru’s [35] research. By 2021, the level of land urbanization in Ningxia Province was relatively high, with a per capita built-up area of 103.40 squares, ranking fourth in the country. The per capita urban road area is 26.23 squares, ranking second; the per capita construction land area is 94.73 squares, ranked 3rd. This shows that the moderate expansion of urban construction land area can improve the level of land use and bring impetus to the development of industrialization, which will further accelerate the process of urbanization. The provinces with low urbanization levels are mainly located in the western region, and the remaining four provinces are Yunnan, Guizhou, Qinghai and Gansu. The average value of new urbanization is less than 0.25, and the level of new urbanization in Yunnan Province is only 0.209. Although it has developed rapidly in recent years, there is a large gap compared with the national average. Yunnan is an underdeveloped area located on the southwest border, and is mostly mountainous and a plateau. In addition, it has been affected by the urban rural dual structure for a long time. The foundation is weak, and the start is late, which is also one of the reasons for the low level of urbanization in Yunnan Province.

From the perspective of the average annual growth rate, China’s urbanization basically presents a pattern of “high growth low level” and “low growth high level”, which further indicates that the urbanization development vitality of high-level provinces is insufficient, while the urbanization development potential of low-level provinces is being released. During the study period, the development trend of the urbanization level in the central and western regions was good, with an average annual growth rate of more than 6.5%. The growth rates of Guizhou and Gansu Provinces are the top two in China, with growth rates of 11.413% and 9.243% respectively. The reason is that the urbanization level of the above two provinces in 2010 was approximately 0.1, and the urbanization starting point was low. Due to the introduction of new urbanization and the two-way drive of reform and innovation, the development potential of urbanization has been fully tapped, to achieve rapid urbanization over 12 years. However, the development trend of the urbanization level in the eastern region is not ideal, and the average annual growth rate of provinces in the region is basically less than 6%. The average annual growth rates of Beijing and Shanghai are less than 3%, at 2.717% and 2.753% respectively, but the urbanization levels of the above areas are greater. This is mainly because the abovementioned areas have a good foundation for new urbanization, but they are highly dependent on the traditional urbanization path, resulting in a higher level of urbanization but a slower development rate.

### Provincial CEECI evaluation

Based on the development stage of China’s CEECI above, the article only lists the calculation results of the key node CEECI (Table 4).

**Table 4:** Calculation results for China’s provincial CEECI

Regional	Province	2010	2013	2014	2018	2019	2021	Average	Average annual growth rate (%)
East	Beijing	0.887	1.043	1.047	1.036	1.062	1.031	1.031	1.375
	Tianjin	0.898	1.011	1.014	0.674	0.627	0.673	0.865	− 2.595
	Hebei	0.704	0.823	0.873	0.672	0.760	0.806	0.763	1.241
	Liaoning	0.821	0.957	0.845	0.612	0.587	0.661	0.723	− 1.959
	Jilin	0.871	0.869	0.977	0.678	0.637	0.748	0.796	− 1.364
	Heilongjiang	0.814	0.922	0.914	0.521	0.500	0.614	0.717	− 2.535
	Shanghai	1.003	0.986	1.004	1.014	1.008	1.103	1.004	0.874
	Jiangsu	1.024	0.958	0.976	1.001	1.025	1.023	0.978	− 0.014
	Zhejiang	1.025	1.013	1.010	0.947	0.955	1.016	1.000	− 0.080
	Fujian	0.773	0.815	0.809	0.869	0.946	0.972	0.857	2.107
	Shandong	0.605	0.699	0.738	0.653	0.697	0.786	0.688	2.404
	Guangdong	0.639	0.763	0.747	0.693	0.725	0.774	0.729	1.759
	Hainan	1.018	1.040	0.882	0.824	0.732	0.803	0.894	− 2.133
Midland	Shanxi	0.641	0.727	0.706	0.672	0.676	0.659	0.668	0.250
	Inner Mongolia	0.680	0.671	0.616	0.501	0.542	0.601	0.606	− 1.119
	Anhui	0.760	0.816	0.822	0.813	0.772	0.883	0.834	1.376
	Jiangxi	0.934	1.001	1.056	0.906	1.006	1.012	0.987	0.740
	Henan	0.718	0.776	0.771	0.760	0.832	0.795	0.773	0.935
	Hubei	0.808	0.922	0.947	1.016	1.044	1.050	0.941	2.416
	Hunan	0.830	1.005	1.009	0.901	0.938	0.981	0.929	1.528
	Shaanxi	0.886	0.881	0.886	0.788	0.776	0.805	0.849	− 0.867
	Chongqing	0.768	0.905	0.961	0.948	0.982	1.172	0.963	3.913
West	Sichuan	0.603	0.754	0.794	0.822	0.819	0.781	0.756	2.372
	Guizhou	0.521	0.633	0.678	0.588	0.591	0.613	0.604	1.495
	Yunnan	0.632	0.774	0.713	0.769	0.779	0.827	0.750	2.472
	Guangxi	0.744	0.885	0.914	0.938	1.009	1.013	0.913	2.852
	Gansu	0.617	0.749	0.723	0.561	0.496	0.523	0.629	− 1.490
	Qinghai	0.710	0.719	0.687	0.581	0.622	0.782	0.661	0.890
	Ningxia	0.807	1.021	0.836	0.728	0.701	0.750	0.812	− 0.672
	Xinjiang	0.875	1.012	1.019	0.682	0.643	0.775	0.865	− 1.096
Nationwide		0.787	0.872	0.866	0.772	0.783	0.834	0.819	0.531

**Fig.5:**
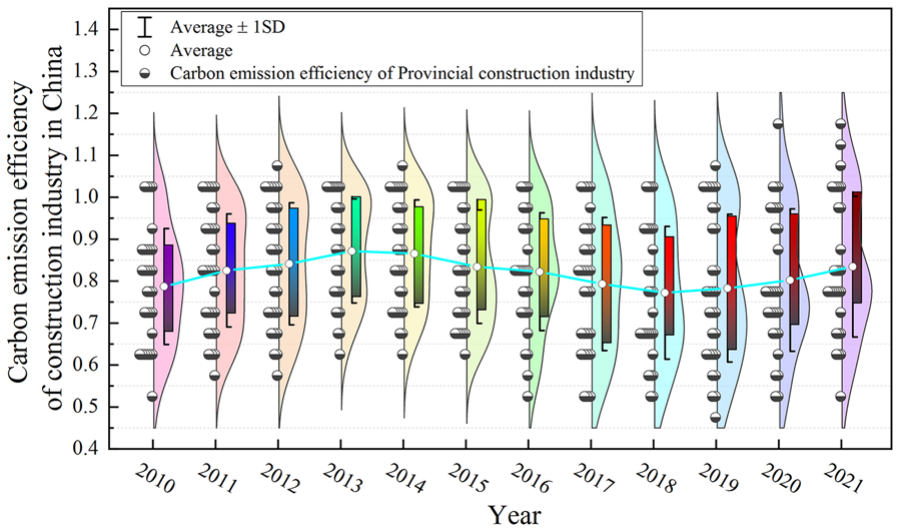
Chinese CEECI violin figure

Figure 5 shows that the national CEECI exhibited a fluctuating upwards trend from 2010 to 2021, indicating that the low-carbon development trend of China’s construction industry was good. The difference between the high value area and the low-value area of the violin diagram gradually become significant. The cabinet of the box line diagram is obviously extended, indicating that the difference in the CEECIs between provinces in China is gradually becoming significant. The trailing at the bottom of the box plot gradually increases, indicating that China's CEECI is greater in the low-value area and that its efficiency is lower.

Specifically, China’s CEECI increased from 0.787 in 2010 to 0.834 in 2021, showing an overall upwards trend, with an average annual growth rate of 0.531%, but it is still not on the production frontier. It is necessary to further improve the carbon emission efficiency of the construction industry. Green technology and zero-carbon buildings should be vigorously developed to promote low-carbon development in the construction industry. The average CEECI value in China is 0.819, and the volatility is strong. The overall development trend is “N type”. The development trend is basically consistent with the results of the existing research [36], which can be divided into three stages. From 2010 to 2013 (the first stage), China’s CEECI showed an upwards trend, with a growth rate of 10.801%. To alleviate the impact of the 2008 financial crisis, the Chinese government increased investment in infrastructure in all provinces, which has enabled the scale of the construction industry to increase rapidly. At the same time, green and low-carbon technologies have begun to be applied in the construction industry, so the CEECI has improved significantly. From 2014 to 2018 (the second stage), the CEECI showed a downwards trend, with a decrease of 11.468%. This may be due to the ability of the country to promote the construction of an ecological civilization, the provinces increasing energy conservation and emission reduction efforts to reduce the investment in the field of construction, and the difficulty of adapting the construction of low-carbon technology to the energy conservation and emission reduction requirements; thus, the construction industry in this region is still hindered from maintaining high carbon emissions. From 2019 to 2021 (the third stage), the national CEECI declined, and the average annual growth rate increased annually. On the one hand, due to the COVID-19 epidemic, the carbon emissions of public buildings in the operation stage have been greatly reduced, so the carbon emissions of the construction industry have decreased; on the other hand, due to the impact of energy conservation and emission reduction policies, China has vigorously developed clean energy such as wind energy and solar energy, replacing high-carbon emission energy and playing an active role in energy structure reform.

**Fig.6:**
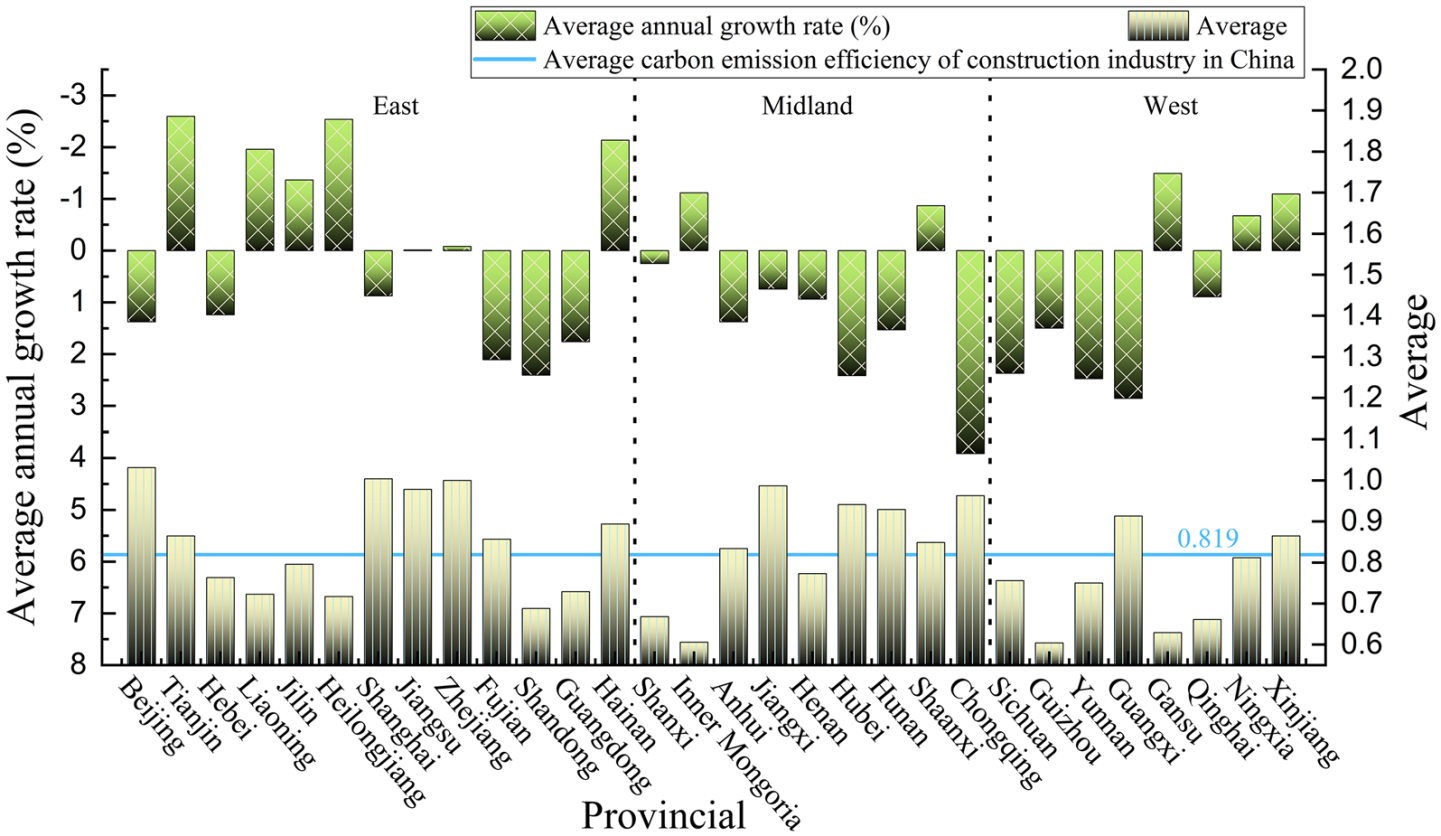
The average and average annual growth rate of the CEECI in China’s provinces

At the provincial level (Fig. 6), China’s provincial CEECI is mostly at a low level, and the uneven development between provinces is significant. There are 18 provinces that achieve positive growth, but only 8 provinces have an efficiency value greater than 1; 12 provinces show a downward trend, of which 3 provinces decrease by more than 2%. This shows that China needs to narrow interprovincial differences and focus on improving the green transformation and development of low-efficiency areas of carbon emissions in the construction industry. There are 15 provinces whose average CEECI value exceeds the national average of 0.819, and most provinces have years with CEECI values above 1. The mean CEECIs in Beijing, Shanghai and Zhejiang are above 1, which is at the forefront of production. This is mainly due to the good economic and social foundation and the high level of urbanization, which allow the construction industry to achieve large-scale development; at the same time, the reserve of scientific and technological talent is sufficient, and the low-carbon technology innovation ability is strong, which provides technical support for the green development of the construction industry. Guizhou Province, Inner Mongolia Autonomous Region and Gansu Province were the three provinces with the lowest CEECIs, with average CEECIs less than 0.63. The efficiency of Guizhou Province is on the rise, but the volatility is strong. Gansu and Inner Mongolia show a downwards trend, and the carbon emission efficiency needs to be further improved, which is consistent with the analysis results of existing research [37]. Specifically, Guizhou and Gansu are located in the western region. Due to their slow economic and social development and lagging urbanization development, it is difficult for the construction industry to undergo large-scale development. Moreover, low-carbon technology and a low energy utilization rate are also factors restricting the low-carbon development of the construction industry. Although the level of urbanization in Inner Mongolia is relatively high, which means that construction needs to increase, there are shortcomings in the green development of the construction industry, and the utilization rate of green technology is low, which makes it difficult to adapt to the requirements of urbanization and energy conservation and emission reduction, resulting in a downwards trend in efficiency.

During the study period, China’s CEECI showed obvious regional differences, mainly reflected in the high carbon emission efficiency in the eastern region, while the distribution characteristics of the CEECI in the central and western regions were low. However, the development trend of the CEECI in the central and western regions is good, and the average annual growth rates of Chongqing and Guangxi are among the top two in China. The reason is that the CEECIs of provinces in the central and western regions were generally low in 2010. Due to the promotion of new urbanization and ecological civilization construction, China has invested more in the coordinated development of the economy and environmental protection, and the emission reduction effect and technical level of the construction industry have significantly improved; thus, the low-carbon development potential of the construction industry has fully tapped. The average annual growth rate of the CEECI in the eastern region is not ideal. Among the 12 provinces with a negative average annual growth in the CEECI, 7 provinces are in the eastern region. On the one hand, the CEECIs of the provinces in the eastern region are a high, and the space for improvement is limited and difficult. On the other hand, the economic level of the eastern region is relatively high; in recent years, with rapid economic development, the economic level of the construction industry and its related industries has also continuously improved, which has had negative effects on the environment. Among them, the Tianjin CEECI has the lowest average annual growth rate. This is mainly because in recent years, Tianjin has carried out active talent introduction work, which has led to a sharp increase in the population, resulting in an increase in construction demand, a rapid expansion of the construction industry, and a low application rate of green low-carbon technologies, resulting in a fluctuating downwards trend in the Tianjin CEECI. Therefore, the effect of improving the CEECI through population urbanization is not ideal.

## Research on the coordination level of urbanization and the CEECI

### Space–time evolution analysis

The coupling coordination degree and relative development model are used to measure the coordination level of China’s provincial urbanization and CEECI from 2010 to 2021, and the average method is used to determine the regional coordination level. The results are shown in Table 5.

**Table 5:** Coupling coordination degree and relative development degree of regional urbanization and the CEECI

Year	Coupling coordination degree				Relative development degree			
	Eestern	Midland	Western	Nationwide	Eestern	Midland	Western	Nationwide
2010	0.454	0.375	0.371	0.408	3.247	4.475	4.539	3.960
2011	0.473	0.391	0.382	0.424	3.030	4.195	4.338	3.728
2012	0.483	0.401	0.392	0.434	2.885	3.919	4.014	3.496
2013	0.494	0.417	0.406	0.448	2.819	3.667	3.799	3.334
2014	0.508	0.431	0.429	0.464	2.687	3.420	3.409	3.100
2015	0.536	0.449	0.444	0.485	2.341	3.146	3.174	2.805
2016	0.550	0.466	0.462	0.501	2.247	2.928	2.876	2.619
2017	0.570	0.481	0.484	0.520	2.055	2.743	2.552	2.394
2018	0.584	0.498	0.501	0.536	1.912	2.555	2.378	2.229
2019	0.596	0.508	0.517	0.549	1.845	2.486	2.217	2.136
2020	0.597	0.516	0.522	0.552	1.885	2.431	2.206	2.134
2021	0.606	0.519	0.525	0.558	1.882	2.417	2.246	2.140

**Fig.7:**
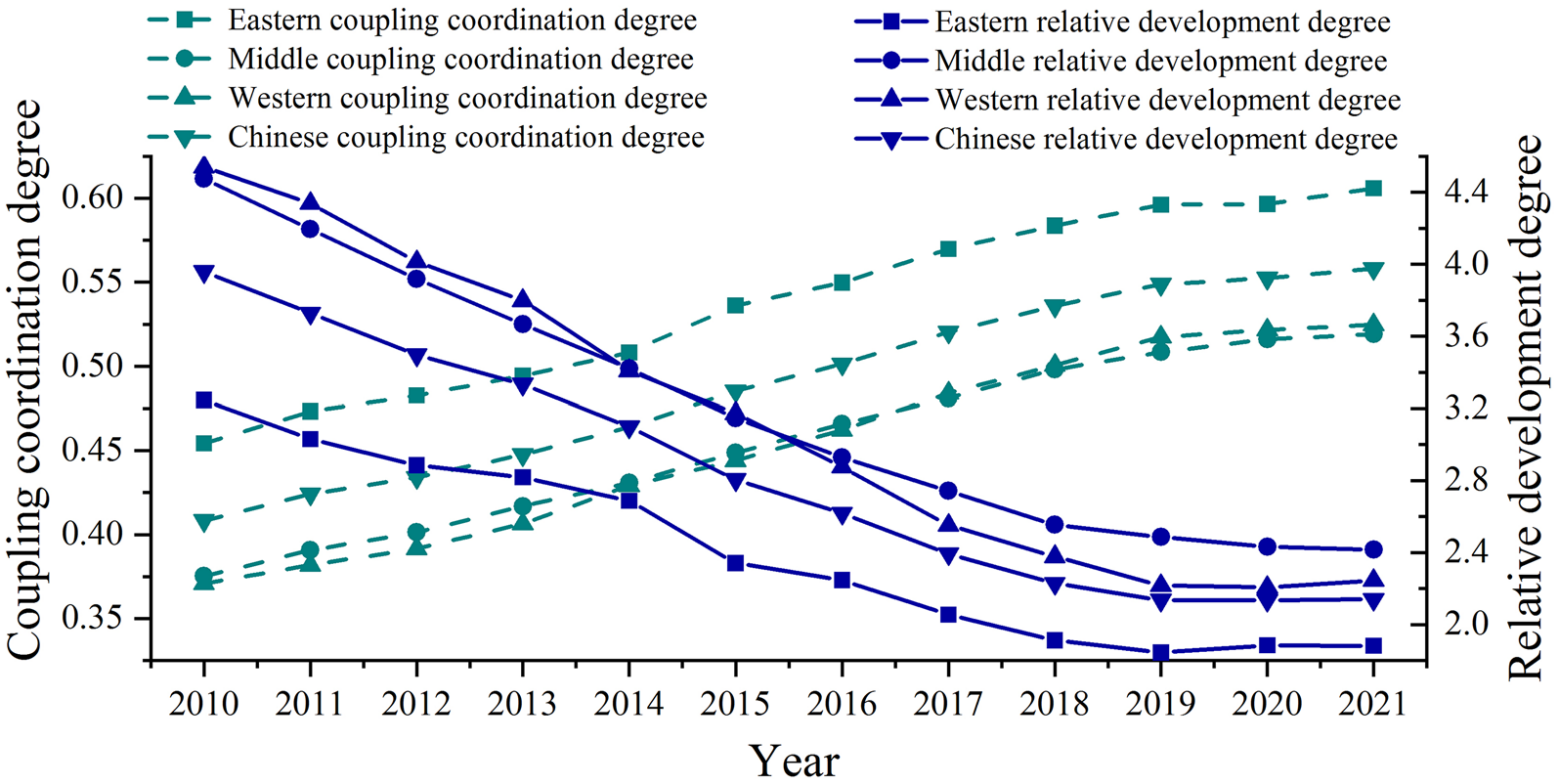
Coupling coordination degree and relative development degree of regional urbanization and the CEECI

As shown in Table 5 and Fig. 7, from 2010 to 2021, the coupling coordination degree of urbanization and the CEECI in China increased annually, from 0.408 to 0.558, an increase of 36.765%; the relative development degree between the two systems showed a downwards trend as a whole, from 3.960 to 2.140, a decrease of 45.960%. Therefore, the coordinated development type of the two systems changes from a low running-in synchronization type to a high running-in efficiency lag type. This shows that in the process of rapid urbanization, due to population agglomeration and the improvement of people’s living standards, the public’s demand for housing and other buildings has increased, promoting the rapid development of the construction industry. At the same time, in the process of rapid development of the construction industry, there are shortcomings in the application of green and low-carbon technologies and large-scale operation, which eventually leads to the two systems being at a high running-in level and showing that the CEECI lags behind urbanization. From this point of view, although the coupling coordination degree between the two systems has increased, it is still in the running-in stage. The stage characteristics are obvious. From 2010 to 2015, the coupling coordination degree increased greatly, while from 2016 to 2021, the coupling coordination degree between the two systems gradually narrowed and basically entered the platform period. This is mainly because during the “Twelfth Five-Year Plan” period, the proposal of new urbanization and ecological civilization construction enabled the two systems to develop rapidly, and the coupling coordination degree of the two systems increased rapidly during this period. However, as the development of each system has entered a bottleneck period, the coupling coordination degree has increased slowly. The relative development degree between the two systems also declined rapidly during the “Twelfth Five-Year Plan” period, and the decline gradually narrowed in the later period; however, the decline in the relative development degree decreased in 2021, showing an upwards trend. This shows that the green development of the construction industry has achieved certain results, and the lack of coordination with the development of urbanization has begun to improve. During the study period, it was difficult for the extensive development of the construction industry to adapt to the accelerated process of urbanization, resulting in the relative development of the CEECI and urbanization from the initial synchronous development to the CEECI lag, reflecting the weak ability of urbanization to drive the low-carbon development of the construction industry. Therefore, when formulating an urbanization development plan, attention should be given to the development of the construction industry, the carbon emission standards of the construction industry should be rationally standardized, and the driving effect of urbanization on the high-quality development of the construction industry should be strengthened to achieve a benign interaction between the two.

From a regional perspective, the development trend of the coupling coordination degree and relative development degree between the two systems in the three major regions from 2010 to 2021 is basically consistent with the national development trend. The degree of coupling coordination between systems in the eastern region is greater than that in the central and western regions, while degree of the relative development is less than that in the central and western regions; therefore, the degree of coordination between systems in the eastern region is always greater than that in the central and western regions. This shows that not only are the levels of urbanization and the CEECI in the eastern region are higher than those in other regions but also that the adaptability between the two is stronger. The coupling coordination degree increased from 0.454 in 2010 to 0.606 in 2021, resulting in the eastern region transitioning from the running-in development stage to the coordinated development stage in 2021. The relative development degree decreased to below 2.8 in 2014 from synchronous development to an efficiency lag. At the same time, the relative development degree rapidly declined in 2015, with a decrease of 12.877%, which may be related to the rapid decline in the CEECI in the eastern region in 2015. During this period, 10 of the 13 provinces in the eastern region experienced a sharp decline in the CEECI. Therefore, the eastern region should pay more attention to the high-quality development of the construction industry and strengthen its demonstration and leading role. The coordination level between the two systems in the central and western regions is not much different, ranging from the low antagonistic urbanization lag type in 2010 to the high running-in efficiency lag type in 2021. During this period, the evolution trend of the coupling coordination degree and relative development degree in the central and western regions was unstable. The coupling coordination degree of the western region exceeded that of the central region for the first time in 2017, and the gap gradually widened. The relative development degree of the central region exceeded that of the western region in 2014, and the gap gradually widened. This shows that the coupling coordination degree in the western region is greater than that in the central region, but the high-quality development of the construction industry has a greater lag. Specifically, the coupling coordination degree of the central region increased from the antagonistic development stage to the running-in development stage in 2012, and the relative development degree changed from lagging urbanization to synchronous development in 2012 and from lagging synchronous development to lagging efficiency in 2017.The coupling coordination degree of the western region changed from the antagonistic development stage to the running-in development stage in 2013, while the relative development degree between systems changed from lagging urbanization to synchronous development in 2013, and from synchronous development to lagging efficiency in 2017.

**Fig.8:**
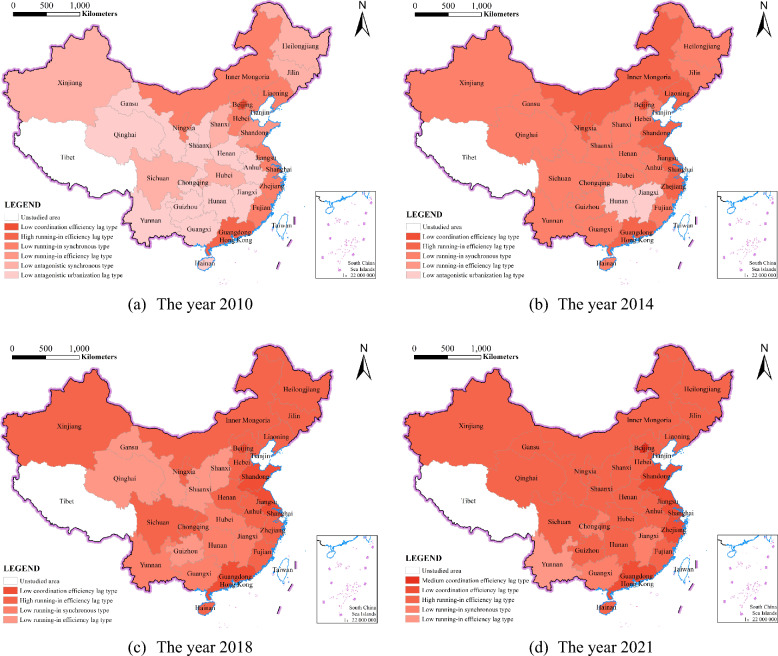
The results of the classification of the coordinated development types of urbanization and CEECIs in China’s provinces

To more intuitively show the differences in the level of coordination between provincial systems, ArcGIS software is used to visualize the types of coordinated development in each province. Figure 8 shows that the coordination level between the two systems in China’s provinces and regions has improved to varying degrees. The degree of coordinated development changed from a low-level antagonistic urbanization lag in 2010 to a high-level running-in efficiency lag in 2021. The number of provinces in the coordinated development stage is increasing annually, from 1 province in 2010 to 7 provinces in 2021. This shows that the provinces have grasped the task of coordinating the development of urbanization and the construction industry for more than 10 years and have achieved certain results. The types of coordinated development of China’s two provincial systems changed from 6 types in 2010 to 5 types in 2021, and the distribution of coordination types in 2021 was more concentrated than that in 2010. This shows that the interprovincial differences are significantly weakened, and the coordination level between the two systems generally shows a distribution pattern of “high in the east, and low in the middle and western regions”.

In 2010, the coordination level between the two systems at the provincial level was quite different, and included three development stages: coordination, running-in and antagonism. Specifically, only Beijing is at the level of coordinated development, which is a low coordination efficiency lag type. This may be because Beijing, as China's political, economic and cultural centre, has high-quality resources, so that its development in all aspects is better than that of other provinces and cities. There are 11 provinces in the running-in stage, of which Shanghai and Guangdong have entered a high running-in level, both showing a state of efficiency lag. As a populous province, the coordination level of Shandong Province is a low running-in efficiency lag type, because its urbanization level is at a high level of 0.281, but the carbon emission efficiency of the construction industry is ranked in the bottom three in the country. This shows that population agglomeration is a weak driving force for the coordinated development of these two systems, and more attention should be given to the low-carbon development of the construction industry. The remaining eight provinces are at the middle level of urbanization and CEECIs in the country, and their development is relatively balanced, resulting in a low level of coordination between the systems. There are 18 provinces with low levels of hostility, 12 of which have lagged rates of urbanization. These provinces include Shaanxi and Chongqing, which are located in the middle and western regions, respectively. Synchronized development was observed in six provinces, namely Xinjiang, Sichuan, Shanxi, Hubei, Heilongjiang, and Jilin. From the perspective of regional distribution, the provinces in the antagonistic development stage are basically in the middle and western regions of China. This is because the economic conditions are relatively backwards, the foundation of urbanization development is weak, and the low-carbon development and large-scale development of the construction industry are insufficient, which leads to the two being in the antagonistic stage.

In 2014, the gap between provincial coordination levels gradually narrowed, and the proportion of provincial areas in the running-in development stage increased from 36.7% in 2010 to 90%, indicating that the quality and balance of provincial development improved; however, only Beijing exhibited a low coordination efficiency lag. There are 8 provinces at the high running-in level. Although the coordination level of Shanghai and Guangdong has improved, it is still a high running-in efficiency lag type. Except for Shandong, the new provinces are promoted from low-level running-in synchronization to high-level running-in efficiency lag, while Shandong is changed from low-level running-in efficiency lag to high-level running-in efficiency lag, achieving a two-level jump. There are 19 provinces at the low running-in level, mainly located in the central, western and northeastern regions of China. Among them, Tianjin has been reduced from a low-level running-in synchronization type to an efficiency lag type. This is because urbanization has increased by 26.01% compared with that in 2010, while the carbon emission efficiency of the construction industry has increased by only 12.92%, which makes it difficult for the low-carbon development of the construction industry to match the speed of urbanization development; the coordination type of the remaining 18 provinces is low-level running-in synchronization. Only Hunan and Jiangxi in the central region maintained a low level of antagonism, and both showed a lagging state of urbanization. On the whole, China has changed from a low antagonism level as the main body to a low running-in level as the main body, indicating that the level of coupling and coordination between the two systems has increased significantly and has shown a trend of spreading from east to west. At present, there are only two provinces in the lagging state of urbanization. This is mainly due to the new urbanization development strategy proposed by the Party Central Committee and the policies on urbanization development issued by each province during the “Twelfth Five-Year Plan” period. As a result, the urbanization development speed is greater than the low-carbon development speed of the construction industry, so that the two systems change from the lagging state of urbanization to the synchronous development or efficiency lag state. Hunan and Jiangxi Provinces should pay attention to their own urbanization construction process, and should make good use of the radiation capacity of the surrounding highly coordinated provinces.

In 2018, the coordination level between China’s provincial systems improved compared with that in 2014. It has changed from a large proportion of low running-in grades to a high running-in grade, and no provinces are in the antagonistic development stage, indicating that the coordination level of the two systems has fundamentally changed. The number of provinces with low-level coordinated efficiency lagging behind increased to 5 with the addition of Shandong, Jiangsu, Shanghai, and Guangdong, all of which are located in the eastern coastal region. This was mainly due to the rapid economic and social development in these provinces, which attracted a large amount of sustainable development talent. This not only contributed to the rapid advancement of new urbanization construction but also allowed the potential for low-carbon development in the construction industry to be realized, thus achieving low-level coordination between urbanization and the CEECI. There were 15 provinces with high running-in levels, showing the characteristics of an efficiency lag. Among them, all the provinces in the eastern region had high-level grinding or above, while the provinces in the central and western regions had high-level grinding. This implies that the central and western regions will rely on the radiation effect of high-level provinces to improve the coordination level between the provincial systems. There were 5 provinces in the low-level coordination synchronization type, among which Jiangxi and Hunan urbanization, with an increase of more than 35%, while the carbon emission efficiency of the construction industry achieved negative growth, thus achieving synchronized development between the two systems. The government needs to work on the green development of the construction industry. Hubei, Yunnan, and Guangxi also achieved rapid growth in both systems. The five provinces of Gansu, Qinghai, and Guizhou in the west and Shanxi and Shaanxi in the central region were in the low-level grinding grade and showed efficiency lagging behind that of the other provinces. There is an urgent need to seize national policy dividends and achieve high-quality coordinated development of urbanization and the construction industry based on their resource advantages and local conditions.

In 2021, the proportion of provinces at a high running-in level increased by 13.3 percentage points compared with that in 2018, and the number of provinces at a low running-in development stage decreased from 10 to 4, indicating that the coordination level between provincial systems further improved. The regions with low coordination levels among the provincial systems in China are mainly distributed in Southwest China, all of which are at a low running-in level, indicating that the difference in the coordination levels among the provincial systems is significantly reduced. The achievements of this stage are mainly due to the government's emphasis on the green and high-quality development of the construction industry and the construction of new urbanization. The Chinese government issued the “National New Urbanization Plan (2014–2020)” and the provinces issued and implemented a number of policies and regulations on the green development of the construction industry and the promotion of new urbanization. Due to the strong support of relevant supporting policies, the low-carbon and large-scale development of the construction industry has deepening in recent years and the level of new urbanization has rapidly improved, so that the level of coordination between the CEECI and urbanization has continuously improved. The number of provinces in the coordinated development stage increased to 7, among which the coordination level between Beijing systems lagged behind the intermediate coordination efficiency. This may be because the continuous promotion of the construction of the Xiong’an New Area has actively alleviated population pressure and the development dilemma of the construction industry in Beijing, thus further enhancing the coordination of the development of the two systems. There are six provinces at the low-level coordination levels. The coordination level of Shandong, Jiangsu, Shanghai and Guangdong have further improved, and two provinces and cities, Tianjin and Zhejiang, have been added. This is because the two provinces and cities have vigorously promoted industrial transformation and upgrading and technological innovation, thus promoting have urbanization and CEECI improvement, and achieving complementary development of the two systems. The number of provinces with high running-in levels increased from 15 to 19 in 2018, and the northern part of China achieved high running-in development of the two systems. The number of provinces at the low running-in level decreased to 4, among which Yunnan decreased from the low running-in synchronization type to the efficiency lag type. Because the urbanization growth rate is 21.57%, which is much higher than the 7.54% of the CEECI, the relative development degree is reduced to below 2.8, indicating that Yunnan needs to make full use of its own ecological advantages to realize the green development of the construction industry. Jiangxi and Guangxi maintain a low-level running-in synchronization, while Chongqing decreases from a high-level running-in efficiency lag to a low-level running-in synchronization. It may be that too much attention is given to the green development of the construction industry and that the promotion of urbanization is neglected; thus, the growth rate of the CEECI is much greater than that of urbanization.

### Analysis of spatial correlation characteristics

#### Global spatial autocorrelation analysis

The degree of coupling coordination between the two systems in the province is related not only to the development of its own subsystems, infrastructure construction and scientific and technological level, but also to the economic development level of the surrounding cities, thus showing a certain spatial correlation. This section uses the global Moran’s I to analyse the global spatial correlation between the two systems in the province from 2010 to 2021. The analysis results are shown in Table 6.

**Table 6:** Global Moran’s I of the coordination level between urbanization and CEECIs in China's provinces

Year	Global Moran’s I/${I}_{g}$	Z-value	P-value*
2010	0.186	1.926	0.027
2011	0.247	2.408	0.008
2012	0.279	2.648	0.004
2013	0.223	2.185	0.014
2014	0.128	1.361	0.087
2015	0.207	2.009	0.022
2016	0.204	2.003	0.023
2017	0.224	2.145	0.016
2018	0.277	2.577	0.005
2019	0.226	2.144	0.016
2020	0.181	1.757	0.039
2021	0.235	2.240	0.013

As shown in Table 6, the global Moran index values of the coordination level between provincial urbanization and the CEECI in China from 2010 to 2021 are all positive, the P values in the 12 years are all less than 0.05, and the test results pass the test at 95%, indicating that the coordination level between the two provincial systems in China presents a positive spatial autocorrelation distribution. That is, the provinces with a “high” or “low” coupling coordination level between the two systems are spatially clustered, indicating that the province with a high coupling coordination level between the two systems has a high coupling coordination level in its neighbouring province, and vice versa. During the study period, the global Moran index of the coupling coordination degree between the two provincial systems in China showed a fluctuating upwards trend, from 0.186 at the beginning of the study to 0.235 at the end of the study, indicating that with the enhancement of interprovincial transportation convenience, the development of domestic market integration, and the enhancement of factor flow degree. The spatial positive correlation between the level of coordination between the two systems in China is increasing. Although the overall Moran index showed an upwards trend, it showed a “V” type fluctuation trend. The index value fluctuated from 0.186 in 2010 to 0.128 in 2014, which may be due to the blind development of the backwards development zone and the increase in factor investment in the province, resulting in the slow flow of basic factors between provinces. From 2014 to 2021, the global Moran index showed a fluctuating upwards trend, rising from 0.128 in 2014 to 0.235 in 2021. Although there were certain fluctuations during this period, it still gradually strengthened overall. With the continuous improvement of transportation infrastructure and the in-depth development of social and economic integration, industrial, cultural and economic exchanges have become more frequent, thus promoting economic development and social progress. The spatial connections between provinces became closer.

#### Local spatial autocorrelation analysis

To reveal the spatial agglomeration characteristics of the coordination level between urbanization and the CEECI in various provinces, this paper uses the local Moran’s I to analyse the local spatial autocorrelation of the coupling coordination degree between the two systems in China's provinces in 2010, 2014, 2018 and 2021. The selection of these four years not only conforms to the temporal variation characteristics of the global Moran’s I as an “inverted V” type but also runs through the “11th Five-Year Plan”, “12th Five-Year Plan”, “13th Five-Year Plan” and “14th Five-Year Plan”, which have certain research significance.

Through the Moran scatter plot, the spatial agglomeration characteristics of the coordination level between the two systems in each province are further analysed. However, due to incomplete image display and other reasons, this paper will use the table form to present the spatial characteristics of the coordination level of each province, as shown in Table 7. From the perspective of different time periods, numerous provinces can be found in the first and third quadrants, indicating that China’s urbanization and CEECI coordination levels have low- and high-value cluster agglomeration distribution characteristics. In other words, the high-high and low-low agglomeration areas of the coordination levels between the two systems in China clearly exhibit spatial convergence characteristics. Over the years, the number of provinces where the coupling coordination level between the two systems was located in the third quadrant was significantly greater than that in the first quadrant, and the distribution in the third quadrant was relatively concentrated, while the distribution in the first quadrant was scattered. This indicates that the number of provinces with lower coordination levels between the two systems is greater than that of provinces with higher levels, suggesting that China’s coupling coordination levels between the two systems exhibit a polarization trend that forms two types of convergence: low-level convergence and high-level convergence. Furthermore, this distribution mode remains largely unchanged as the coupling coordination levels between the two systems improve because the coupling coordination level between the two provincial systems in China presents a trend of polarization, forming two types of convergence, namely, high-level convergence and low-level convergence, and this distribution pattern has not changed fundamentally with the improvement of the coupling coordination level between the two systems. The number of provinces in the second and fourth quadrants of the Moran scatter plot in 2021 was greater than that in 2010, indicating that the spatial heterogeneity of the coupling and coordination levels of the two systems in China gradually increased.

**Table 7:** Spatial distribution of coupling coordination level between urbanization and CEECI in China’s provinces

Year	First Quadrants	Second Quadrants	Third Quadrants	Fourth Quadrants
2010	Beijing, Tianjin, Shanghai, Jiangsu, Zhejiang, Shandong, Guangdong, Fujian	Inner Mongolia, Chongqing	Shanxi, Jilin, Heilongjiang, Anhui, Jiangxi, Henan, Hubei, Hunan, Guangxi, Hainan, Sichuan, Guizhou, Yunnan, Shaanxi, Gansu, Qinghai, Ningxia, Xinjiang	Hebei, Liaoning
2014	Beijing, Tianjin, Shanghai, Jiangsu, Zhejiang, Shandong, Guangdong, Fujian, Inner Mongolia, Liaoning	Hubei, Chongqing	Hebei, Shanxi, Jilin, Heilongjiang, Anhui, Jiangxi, Henan, Hunan, Guangxi, Hainan, Guizhou, Yunnan, Shaanxi, Gansu, Qinghai, Ningxia, Xinjiang	Sichuan
2018	Beijing, Tianjin, Shanghai, Jiangsu, Zhejiang, Shandong, Inner Mongolia, Guangdong	Fujian, Chongqing	Shanxi, Jilin, Heilongjiang, Anhui, Jiangxi, Hunan, Guangxi, Hainan, Guizhou, Yunnan, Shaanxi, Gansu, Ningxia, Xinjiang	Liaoning, Henan, Heilongjiang, Sichuan
2021	Beijing, Tianjin, Shanghai, Jiangsu, Zhejiang, Shandong, Guangdong	Inner Mongolia, Fujian, Chongqing, Qinghai, Hebei	Shanxi, Jilin, Heilongjiang, Anhui, Jiangxi, Hunan, Guangxi, Hainan, Guizhou, Yunnan, Shaanxi, Gansu, Ningxia, Xinjiang, Heilongjiang	Liaoning, Henan, Sichuan

### Coordinated development optimization path

The coordinated development of urbanization and the CEECI is an inherent requirement for achieving China’s “double carbon” goal and high-quality development. The results show that there are great differences in the trends of urbanization and the CEECI in different provinces of China and in the level of coordination between them. The variability of the coordination types and spatial characteristics of provincial urbanization and the CEECI lead to different development pressures, potentials and driving forces faced by different provinces. Therefore, in the future, according to the development goals of urbanization and the low-carbon construction industry, combined with their own realities and problems, provinces should get out of follow the differentiated high-quality coordinated development path of the urbanization and construction industry.

1. Medium coordination efficiency lag type: only China’s capital, Beijing. The coordination degree between urbanization and low-carbon development in the construction industry in Beijing is high, but the CEECI is low. Therefore, as a political, economic and cultural centre, Beijing should take full advantage of its geographical advantages; based on the rich scientific research institutions in Beijing, we will deepen the integration of production, teaching, research and application to more effectively retain talent and ensure innovation; at the same time, we should make full use of the Xiong’an New Area to alleviate the pressure of population and construction industry development in Beijing. While ensuring the high-quality development of urbanization, we will accelerate the optimization of resource allocation in the construction, build a green whole industry chain, and promote the large-scale development of construction enterprises. As a leading area for the coordinated development of urbanization and low-carbon construction industry, it is necessary to play a leading role in bridging the regional development gap through the establishment of interregional cooperation, mutual assistance and support mechanisms.
2. The low coordination efficiency lag type is mainly located in eastern China. The eastern coastal areas are experiencing rapid economic development and more international exchanges, and the levels of urbanization and the CEECI are higher. Therefore, it is very important to balance the relationships among economic development, urbanization and carbon emissions. First, urban renewal should focus on gradual progress, preventing large amounts of demolition and construction while improving building life and improving the quality of urbanization. Second, the scope and coverage of building energy policies and regulations should be expanded, stricter building energy conservation standards and norms should be adopted, the potential for change in relevant departments of the construction industry should be released, and building energy transformation should be achieved. Finally, development should be coordinated according to the actual situation of eastern China, and the key is the integrated development of the Yangtze River Delta region. Cities in the Yangtze River Delta region encourages and supports the development of green buildings, including the use of energy-saving materials, efficient energy systems, and green construction technologies. Green buildings can not only reduce energy consumption and carbon emissions, but also improve living comfort and building life. With reasonable urban planning and design, the layout and form of buildings can be optimized, the utilization efficiency of urban space can be improved, and resource waste and carbon emissions can be reduced. By improving the CEECI, the development potential of urbanization is further activated, and the development adaptability of the two is enhanced. At the same time, Guangdong, Shanghai and other provinces should use their own radiation to drive the transformation and upgrading of surrounding provinces.
3. There are strong running-in efficiency lags, and a large number of provinces, and the coordination level between this province and the surrounding provinces is low. This type of province should actively learn from the experience of the coordinated development of the two systems of benchmark provinces, and should actively cooperate with them to improve their own coordination level through the dissemination and radiation of neighbouring provinces’ experience and technology. First, we should drive the population and industrial agglomeration with the development of urbanization so that the construction industry can develop on a large-scale to enhance the CEECI. At the same time, we should vigorously cultivate and introduce innovative planning and construction talent, promote the extension of urban infrastructure to rural areas, and realize the balanced development of urban and rural areas. Second, the optimization of the energy consumption structure is conducive to improving the carbon emission efficiency of the construction industry. Due to the strong dependence of the construction industry on traditional energy, the price of clean energy such as electricity and natural gas can be adjusted and zero-carbon heating and other methods can be used to improve the interaction between building electricity and the power grid, promote building electrification, and cooperate with the power sector for decarbonization. Finally, through the publicity and guidance of the concept of energy conservation, a social atmosphere advocating green life is created to improve the carbon emission efficiency of the construction industry from the consumer side.
4. The low running-in synchronous type is located in Jiangxi, Chongqing and Guangxi in southern China. Although the two systems in the three provinces are characterized by synchronous development, the CEECI level is higher and the urbanization level is lower. Therefore, it is necessary to further promote high-quality urbanization with people as the core. Located on the south bank of the middle and lower reaches of the Yangtze River, Jiangxi Province has abundant water resources and hilly landforms, which are suitable for the development of green buildings and energy-saving technologies. Through policy guidance to formulate and implement a series of green building standards and codes, new buildings are required to meet certain energy conservation and emission reduction standards. At the same time, the government can introduce a series of supporting policies, such as tax breaks, subsidies, and green credits, to encourage enterprises and individuals to invest in and adopt green building technologies and products. Chongqing has a unique urban planning and architectural style. This terrain requires building design and construction to address energy conservation and environmental protection. With the development of detailed energy efficient building codes and standards, all new and renovated buildings must meet a certain level of energy efficiency, such as the use of energy-efficient materials, the design of efficient lighting and ventilation systems, and the use of renewable energy sources. The building energy efficiency evaluation system should be improved by grading and publicizing the energy efficiency of buildings and guiding consumers and investors to choose high energy efficiency buildings. Guangxi is rich in national culture and ecological resources, so the development of urbanization needs to balance cultural protection and ecological balance. Urbanization development should follow ecological principles to protect and restore the integrity and function of natural ecosystems. This includes rational planning of urban green spaces and water systems and promoting green buildings and sustainable building materials. While promoting economic development, we must also ensure social justice, cultural diversity and ecological health.
5. The low running-in efficiency lag type is located in the southwestern Yunnan. Yunnan’s economy is relatively under developed and its population is sparse, which results in a low level of urbanization and a low level CEECI. Improving the level of urbanization in low-level urbanization areas is conducive to improving the carbon emission efficiency of the construction industry [37]. Therefore, it is necessary to further promote high-quality urbanization with people as the core. First, the role of population urbanization in improving the overall carbon emission efficiency of the construction industry is limited. When the policy orientation is to reduce emissions and improve efficiency, we should not only consider population urbanization but also pay attention to other dimensions of urbanization. Second, through the division of labour and cooperation, regional specialization and economic activity agglomeration are occur, and the positive effect of spatial urbanization on the carbon emission efficiency of the construction industry is brought into play. Ultimately, as Yunnan Province is rich in natural resources and has diverse ecological environments, a green building evaluation system and incentive policy suitable for the region should be formulated with reference to advanced green building standards at home and abroad. We should vigorously develop the green economy, optimize the business environment, encourage digital empowerment, form new economic growth points and new growth drivers in green and low-carbon fields, and provide technical support for urbanization and carbon emission reduction in the construction industry.

## Discussion

The coordinated development of provincial urbanization and the CEECI has important practical significance for achieving high-quality development in China. There are some differences in the level of urbanization and CEECI in China’s provinces. The proposal of the “double carbon” goal is both an opportunity and a challenge to realize the high-quality coordinated development of the two. From the results of this study, it can be seen that the two systems in China’s provinces are still dominated by the high running-in efficiency lag type, only Beijing has entered the medium coordination efficiency lag type, and there is still a great deal of demand for coordinated development of the two systems. China is in a transition period from the running-in stage to the coordination stage. To optimize the coordinated development path of the two, we need to consider the differences and emphases. In reality, we should consider the economic development degree, natural foundation and difference in the development ability of the two provinces. This finding is consistent with those of Lin [37] and Niu [38] et al., who proposed the carbon emission efficiency of the construction industry and the coordinated development of urbanization.

In short, this paper analyses the degree of coordination between urbanization and the CEECI, which is an extension of the research content of the relationship between the two. The existing research mainly studies urbanization as a factor affecting CEECIs, and this paper incorporates the two into a unified framework, using a combination of quantitative and qualitative analysis methods to deeply explore the coordination relationship between the two, which not only enhances the scientific accuracy of the research but also extends the breadth and depth of relevant theoretical research. From a theoretical point of view, the coupling coordination model can effectively describe the dynamic relationship between the coordination level of urbanization and the CEECI and judge whether the low-carbon development of urbanization and the construction industry is in a coordinated state in a timely manner, which provides a new method for researchers, government decision-makers and the public to study the benign interactive development of regional urbanization and the construction industry. From a practical point of view, the methods and conclusions of this paper are applicable to China and provide valuable decision-making references for the design of construction industry decarbonization policies in India, Russia, Iran and other countries [39]. However, this paper still has limitations. (1) Due to the difficulty of data acquisition, this paper takes the province as the research object. In the future, it is necessary to further refine the measurement of the coordination level of the two systems in the city or county, which has more practical value for promoting the high-quality coordinated development of the urbanization and construction industry. (2) In the process of calculating the carbon emissions of the construction industry, because the data are difficult to obtain, the carbon sequestration effect of the green vegetation around the building is not considered, so the carbon emissions of the construction industry may be too large, which leads to deviations in the measurement of the carbon emission efficiency of the construction industry (Additional files 1, 2).

## Conclusion

By establishing a comprehensive evaluation index system of urbanization and the CEECI in 30 provinces in China, this paper uses the improved entropy method and coefficient of variation method to comprehensively measure the level of provincial urbanization, avoiding the drawbacks of the single weighting method and subjective weighting method; The unexpected output superefficiency Windows-EBM-DEA model is used to evaluate the provincial CEECI, which avoids the disadvantages of traditional radial and nonradial models and makes the CEECI comparable in the vertical direction. The coupling coordination degree model with the relative development index is used to evaluate the coordination level between urbanization and the CEECI. Finally, combined with the spatial autocorrelation analysis model, the spatial correlation characteristics of the coordination level of the two systems are analysed. The study revealed the following:

1. China’s urbanization level increased steadily from 2010 to 2021, but the year-on-year growth rate showed a fluctuating downwards trend, indicating that China's urbanization development achieved certain results. There is a significant difference in the urbanization level among provinces. The urbanization level of provinces in the eastern region is higher, but the growth rate is slower. This is due to the high economic and social level of provinces, which leads to a bottleneck in urbanization development and makes rapid improvement difficult. Although the level of urbanization in the western region is low, the growth rate is fast due to the western development policy.
2. During the study period, China’s CEECI exhibited a fluctuating upwards trend, indicating a good trend of low-carbon development in China's construction industry. The CEECI generally presents an “N” type development trend, and inflection points appear in 2013 and 2018. With the promotion of new-type urbanization and ecological civilization construction, the development of the CEECI in central and western China is good, but the efficiency level is low due to the lack of large-scale development and other reasons. However, the CEECI level in the eastern region is high, but the growth momentum of the construction industry is insufficient, and the average annual growth rate is not ideal.
3. During the study period, the coupling coordination degree between China and regional urbanization and the CEECI showed an upwards trend, while the relative development degree showed a downwards trend. The coordination level among the three regional systems improved to varying degrees. By 2021, the three regions have shown an efficiency lag, and the eastern region first entered the stage of coordinated development in 2021. The degree of coupling coordination between the two systems showed the pattern of eastern > middle > western, while the degree of relative development between systems changed from western > middle > eastern to middle > western > eastern.
4. The coordination level of the two systems in each province increased significantly, and the spatial distribution pattern exhibited a state of dynamic change. The overall distribution pattern is “high in the east, low in the middle and western regions”. The interprovincial differences are significantly weakened. By 2021, 63.33% of the provinces are at a high running-in level, and 20% of the provinces in the east have entered the stage of coordinated development, of which only Guangdong has entered the medium coordination level. In addition, 93.33% of the provinces exhibit an efficiency lag.
5. During the study period, the coordination level between provincial urbanization and the CEECI showed a positive spatial autocorrelation distribution, the global Moran index showed a “V”-shaped trend, and the spatial dependence of the coordination level between the two systems gradually weakened. In the local spatial distribution, the coordination level of the two provinces and regions has formed two convergence types: high and low. In addition, the number of provinces located in quadrants II and IV in 2021 increased compared with that in 2010, and the spatial heterogeneity of the coordination level of the two systems gradually increased.
6. According to the five types of coordination between provincial urbanization and the CEECI, namely, the medium coordination efficiency lag type, low coordination efficiency lag type, high running-in efficiency lag type, low running-in synchronous type and low running-in efficiency lag type, from the perspective of urbanization and low-carbonization development in the industry, the corresponding differentiated coordination optimization path is discussed.

### Supplementary Information

**Additional file 1.** Dataset 2.EXCEL is the data processing result of the article.

**Additional file 2.** Dataset 1. The original data of the article EXCEL.

## Data Availability

The dataset supporting the conclusions of this article is included within the article.
